# Community health systems and priority setting for elderly healthcare services in rural Tanzania: Experience from Nzega and Igunga districts

**DOI:** 10.1371/journal.pone.0321482

**Published:** 2025-04-15

**Authors:** Malale Tungu, Nathanael Sirili, Amani Anaeli, Gasto Frumence

**Affiliations:** Department of Development Studies, School of Public Health and Social Sciences, Muhimbili University of Health and Allied Sciences, Dar es Salaam, Tanzania; Noble Shivapuri Research Institute, NEPAL

## Abstract

**Introduction:**

In the 1990s, Tanzania adopted health sector reforms with the aim of engaging the local communities in priority setting and decision-making for effective and efficient use of resources. Community engagement aims to enhance community voice and efficiently allocate available resources according to the citizen’s demands to achieve the targeted health outcomes. The Community Health Systems (CHS) aim to strengthen Primary Health Care (PHC) services by empowering all community actors close to and serving community members. This study explored the role of the CHS during priority setting process in improving health services for the elderly in rural Tanzania.

**Methods:**

An exploratory case study design was employed to collect data using Key Informants Interviews (KIIs) in Nzega and Igunga districts. Purposeful sampling was used to select participants from the two districts. Twenty-four (12 from each district) interviews were conducted with community representative members of the Health Facility Governing Committee (HFGC), social welfare, Council Health Management Team (CHMT), District medical officers, Medical Officers in-charge (MOI), planning officers and health system information focal person. All audio recorded interviews were transcribed verbatim. The transcribed interviews were translated from Kiswahili to English. The data were analyzed using the content analysis approach. The transcribed data, field notes, and documents were reviewed and read to identify broad areas in which to form initial codes and codes. Similar codes with related concepts were grouped to form initial categories and categories.

**Results:**

The findings of this study demonstrated the importance of CHS in strengthening community participation in identifying the elderly who are in need in the community and been involved in elderly matters during priority setting of the elderly health services through the health facility governing committee. This means that there was community participation in elderly matters especially to help the elderly reach health facilities and during priority setting, positive and negative perceptions among community members about the elderly agenda during priority setting. In addition, the findings show that there is poor awareness among community members including family members who perceive that the government is responsible for providing health services to the elderly and not the community or family members.

**Conclusion:**

The findings of this study indicate the importance of community during the priority setting process which plays a great role in identifying the elderly who are in need and the most needed health services for the elderly in their communities. Therefore, the Local government authority should fully involve CHWs in collaboration with all community actors to address elderly matters in rural areas and improve elderly healthcare services. The community members have to be educated and raise awareness about elderly health matters through different platforms such as during world elderly day, village meetings and at the health facility level.

## Introduction

Globally, community participation in health systems during priority setting has a crucial role, especially in less developed countries where most of the governments fail to provide adequate and quality healthcare services for their people [1–3]. Community health systems have been a central theme in most world health-related discussions which is in the World Health Organization (WHO) constitution, confirmed in the Alma-Ata Declaration whereby people are urged to participate individually and collectively in the planning and implementation of their healthcare services [4–6]. Tanzania and other developing nations adopted health sector reforms in the early 1990s. Among other aspects, the reforms aimed to engage the local community in the priority setting and decision-making for effective and efficient use of resources [7,8]. The community participation is aimed at enhancing community voice, empowering marginalized groups including the elderly, efficient allocation of the available resources as per citizen’s demand, improving health service and subsequently improving the quality of healthcare at the lower levels [1]. Part of these reforms was decentralization by devolution whereby the districts were regarded as the focal point for the planning and implementation of all health programs which combine all local levels including the health facility level, community level, village and ward level [9,10]. Decentralization by devolution focused on improving community-level health services as a key component of overall health systems strengthening [8,10,11]. The current idea of CHS aims to strengthen Primary Health Care PHC) services by empowering Community Health Workers (CHWs) towards Universal Health Coverage (UHC) [12].

In Tanzania, community participation is considered a significant dimension in healthcare planning and priority setting for decision-making within CHS under decentralized healthcare systems across the country. The government of Tanzania is pushing accountability for the planning and distribution of health services closer to the communities. Civil Society Organizations (CSOs) and community actors are being asked to play a greater participatory role in these processes of priority setting and planning by considering vulnerable groups including the elderly [13]. The country extended PHC services at the grassroots level by expanding frontline health facilities and training CHWs to conduct community outreach [14].

Evidence from previous studies have presented empirical evidence on the importance of priority setting in local governments through community participation. Decentralization has the reputation of building a relationship between policy-makers at the district level and the local levels through the prioritization process that starts from the community level. The Local Government Authorities (LGAs) aimed at increasing the participation of the community including representatives of special groups such as the elderly, women, youth, disabled, and other stakeholders in setting priorities for healthcare services. Studies show that there is no full participation of the community and other stakeholders in priority setting at the district level [13,15]. In principle, the priority setting process should be based on the guiding principles and criteria including the burden of diseases, magnitude and severity of the problem for equitable distribution of the available scarce resources. However, the process of priority setting is sometimes driven by the historical allocation of mobilized resources at the district level, which lacks appeals mechanisms [15]. This implies that the allocation of resources is based on what was allocated in the previous financial years regardless of the reality of the contemporary dynamics.

District councils identify the priorities and plan how the mobilized scarce health resources can be allocated for health services to meet local health needs [16]. The Council Health Management Team (CHMT) is responsible for planning, implementing, monitoring, and evaluating healthcare services at the council level. The teams perform their responsibilities, by involving different stakeholders at every stage of planning, and budgeting. This ensures that various priorities from the community are incorporated into the planning process and subsequently included in the budget. Decentralized health governance aims to engage communities (through representatives) in council health planning, budgeting, and monitoring processes.

The CHS is a set of different local actors and processes engaged in producing, advocating for, and supporting health in communities and households outside the formal health system [17,18]*.* Community Participation is a function of different groups including public health officials, local organizations, community health leaders, private sector providers, civil society, and frontline workers who are central in identifying and addressing healthcare issues within their areas [19].

Tanzania is among developing nations in which priority setting in healthcare delivery systems is important in ensuring the proper allocation of scarce resources for the provision of responsive health services. Priority setting is the process of formulating systematic rules to decide on the distribution of limited healthcare resources among competing programs or patients [20]. Priority setting focuses on either health or utility depending on whether the decision-maker believes that resource allocation should depend only on the utility obtained by individual people (i.e., welfarism) or depend on factors other than a utility like health (i.e., extra-welfarism) [21].

In priority setting, welfarists explain that the goodness of any state should be judged based on the utility level attained by individuals. Extra-welfarists believe that health outcomes should be more relevant than utility in assessing policies in the health sector [22]. This means that the priority setting process should not consider only the individual demand, but the need for health systems for the allocation of resources in the health sector by considering different criteria [23–26]. The stated criteria include the magnitude of the problem (proportion of people affected by a problem), severity or danger of the problem (how serious the condition is), vulnerability to intervention, equity issues, acceptability by the targeted consumers, the cost of the intervention compared to the health outcomes and political will of the intervention.

Proper priority setting is compelled to ensure financial protection for vulnerable groups including the elderly population. In Tanzania, most of the elderly live in rural areas with poor health services, high rates of chronic diseases and poor economic conditions. It is therefore imperative that the process of priority setting is obliged to take into consideration community members with special attention to the elderly group. Studies on the role of CHS during priority setting process at the district level are scanty and not specific to the improved healthcare services of the elderly in rural areas. Most of them are based on the priority setting process regarding accountability for reasonableness from the experience of the CHMT and decision-makers at the district level [15], actors and contextual factors for healthcare priority setting at local levels [27], priority setting process for family planning, maternal, new-born and child health at the decentralised health system [16]. Other studies on elderly health status but not specific for the role of CHS in priority setting process to improve the health services for the elderly [28–30]. This study therefore aimed at exploring the role of the CHS during the priority setting process in improving health services for the elderly population in rural Tanzania.

## Methods

### Study design

An exploratory case study design using Key Informants Interviews (KIIs) was employed in this study. A case study using two districts of Nzega and Igunga was found appropriate in understanding how the community is being involved in setting priorities regarding health services, particularly for the vulnerable population including elderly people. In other words, this design enabled the research team to explore contextual issues regarding priority setting at the community level. Additionally, considering that priority setting is a complex phenomenon involving social processes, the case study approach was found relevant for this study [31].

### Study setting

This study was conducted in Nzega and Igunga Districts located in Tabora region. The two districts were the relevant districts for a case study in Tanzania since they have most of the rural settings with scattered households. According to NBS [32,33], the total population in Nzega District was 502,252 in 2012 and 699,691 in 2022 while in Igunga was 399,727 in 2012 and 546,204 in 2022. Among these populations, about 6 per cent and 5 per cent of the total population are the elderly above 60 years and above in Nzega and Igunga districts, respectively which is almost the same as the nation of 5.7 per cent. Among the elderly population in Nzega and Igunga districts, about 72 per cent live in rural areas [34]. The elderly in rural areas are characterized with poor health and limited resources to meet healthcare costs. This was among other factors considered in this study, which was conducted in Nzega and Igunga districts to explore the role of the CHS during priority setting, especially in the rural areas where the majority of elderly people are living.

### Study population and sampling procedures

The study adopted purposive sampling in selecting participants for data collection. We purposively selected Key informants who were responsible for the priority setting process from the community to the council level. The study population were purposefully selected based on their day-to-day roles in the priority setting process for elderly health services in the community. If the cadre had more than one participant, the one who was in charge was selected to represent the others in the department. For each district, the following participants were selected: 1 District Planning Officer (DPLO), 1 District Medical Officer (DMO), 1 Medical Officer In-charge (MOI), from the District Hospital 1 Health Management Information System (HMIS) focal person, 1 District Social Welfare Officer (DSWO), 2 Health Facility Governing Committee (HFGCo) members, 1 District Health Secretary (DHS), 1 Hospital Secretary (HS), 1 Council Health Service Board (CHSB), and 2 members from Council Health Management Team (CHMT). This makes 12 participants from each district with an overall total of 24 participants from the two districts. Among the 24 participants, 7 were female and 17 were male, all of whom had experience in the role of CHS during the priority setting to improve health services among the elderly. The community representatives from HFGCo and CHSB represented the CHS.

### Data collection

This study used the Key Informant Interview (KII) guide to explore information on the role of the CHS in the planning process and priority setting to have improved health services for the elderly in rural areas from the study participants. We started by reviewing the documents to familiarize ourselves with the decision-makers on the role of CHS in priority setting and planning. The participants were asked about their experience with the role of the CHS during the priority setting process in improving health services for the elderly in rural Tanzania. The topics covered included the participation of the community in the priority setting related to elderly matters, community perceptions and awareness about the elderly, and the role of CHWs in addressing elderly matters within the community. Key informant interviews were conducted between July and August 2020. Four researchers (whereby two were faculties and two were Master’s Graduates) conducted the interviews. The faculties facilitated the interview while others facilitated the audio recording of the interviews, taking notes on the key themes, asking additional questions and monitoring any interaction. After attaining information saturation of the categories since there was no new information that was coming and thus stopped data collection at 24 respondents, 12 from each district. Study participants were purposively selected based on their experience and role in priority setting from the community to the district level. The key informant interviews with participants working in district councils were conducted in their offices, while others were conducted in selected offices suitable for the interview. All KII were conducted in the Kiswahili language which is widely understood by the majority of ordinary Tanzanians. All KII were audio-recorded using a digital voice recorder and the duration of the interview ranges from 50 to 74 minutes. The research team included a moderator and assisted by a note taker who recorded in the notebook all important issues that emerged during the interview. Debriefing sessions were conducted after every interview for consistency, quality control and capturing new information. In addition, to ensure the trustworthiness of the findings, the study included many participants including members of the different committees, whereby most of them are part of the community based on their experience in the priority setting process at the local level. This study is transferable due to the detailed background data and in-depth methods description to ensure the reliability of the study for further researchers to replicate the study.

### Data management and analysis

All audio recorded interviews were transcribed verbatim. The transcribed interviews were translated from Kiswahili to English. Data were analyzed using the content analysis approach. This approach is used to determine the presence of concepts within texts or a set of texts which limit bias. The approach helped to develop categories from the text data inductively for capturing the experiences of the participants. In addition, this approach entails the interpretation of the content of text data through a systematic classification process of coding and identifying themes or patterns [35,36]. Using the NVivo software, the transcribed data, field notes and documents were carefully reviewed and read to identify broad areas to form initial codes and codes. Similar codes with related concepts were grouped to form initial categories and categories.

### Ethics approval and consent to participate

Ethical clearance was obtained from the Muhimbili University of Health and Allied Sciences (MUHAS research review board) in June 2020 (MUHAS-REC-6-2020-288). Permission for data collection in Tabora region was granted by the Regional Administrative Secretary. Permission for data collection was granted by the District Executive Directors of the Igunga and Nzega districts. Participants were duly informed of the purpose of the study and their rights. Written informed consent for this study that includes data collection and consents to participate was requested and obtained from the participants and they were assured of their anonymity in publications.

## Results

The summary of the study findings is presented in Table 1 which indicates the content analysis process for the categories. The findings are structured into five categories, namely; community participation in elderly healthcare matters, community participation in the priority setting on elderly healthcare matters, community perceptions about the elderly agenda, community awareness about the elderly and less involvement of the CHWs in the elderly healthcare services in the community. This study involved 24 (7 female and 17 male) respondents who are involved in making decisions and setting priorities at the district level.

**Table 1:** Content Analysis process for the categories.

S/N	Sub-categories	Categories
**1**	Community involvement in elderly matters: Helping the elderly to reach the health facility where necessary especially CHWs and other community members. Provide information about the elderly, i.e., contribute a little amount in the community for the elderly who are poor.	Community participation in elderly healthcare matters
**2**	The role of community participation in priority setting: Identification of the elderly ◦ Provide information about the available elderly living with difficulties through CHWs. Helps to promote democracy and transparency during identifying the elderly. Helps to provide data on the most problems which affect more elderly in their locality.	Community participation in the priority setting on elderly healthcare matters
3	Different perceptions from the community, family members, experts, and decision-makers about elderly matters during priority setting. Positive/negative view about elderly agenda: Elderly are important in priority setting because they have vision and are wise. Sometimes the community including family members think that the government is responsible for providing health services to the elderly. Moral issues among family members about the elderly and negative community perceptions about the elderly. ◦ Sometimes perceive them as witchcrafts	Community perceptions about the elderly agenda
4	Unclear information from the community about the responsibilities of the government regarding elderly healthcare services CHWs are less involved in elderly matters (are there for < 5 years and pregnant women) Unclear information about the elderly on accessing free health services including medicines at the health facility.	Community awareness about the elderly
5	CHWs are not much involved in elderly matters If CHWs were to be well utilized in the elderly matters, they could be very important in the improvement of elderly health services, especially in rural areas. Normally CHWs are paid or compensated by other different development partners and not the government.	Less involvement of the CHWs in elderly healthcare services in the community.

### Community involvement in elderly healthcare matters at the community level

The findings of this study show that community members are involved in various issues concerning elderly healthcare matters. These include helping the elderly to reach the health facility where necessary, providing information about the elderly who are in need, and sometimes contributing an amount of money especially for the elderly who are poor to cover some basic expenses for their health.

*“At the village or community level, some elderly individuals are being assisted by their neighbors to reach the health facilities …sometimes the neighbors or community take the responsibility to cover some costs to buy medicines or transport, especially to the elderly who are in need” –(KI#12).*

### Community participation in the priority setting process on the elderly healthcare matters

Community members play a vital role in the priority setting process during planning for the health services at the lower level including elderly healthcare services through the health facility governing committee. This includes the identification of the elderly who are supposed to be prioritized, providing data on the common problems which affect the elderly in their localities and providing information about the availability of the elderly who are living with difficulties in their localities. Further, the community helps to promote democracy and transparency during identifying the elderly since the process is done at different gatherings and meetings at their localities.

*“The community plays a crucial role in the priority setting process, as it initiates at the community level and progresses through the village to the district level. Therefore, the community play a significant role in identifying the elderly individuals eligible for waivers and exemptions, as well as providing information on health problems affecting the elderly in their community…therefore, the elderly matters are recognized at the community level since the elderly are the community…” -(KI#9).*

### The community perceptions of the elderly agenda

The participants reported that there is the existence of different perceptions about the elderly agenda during the priority setting process which may affect positively or negatively the provision of health services to the elderly. Some of the community members recognize the importance of the elderly in the community for their vision and wisdom. On the other hand, some of the community and family members perceive the elderly as not an important group to be prioritized over other groups like under 5 years children and pregnant women. In many cases, some of the community members perceive that most of the elderly people are witches. In addition, sometimes the community including family members perceive that the government is responsible for helping the elderly and not the community or family members.

*“…It is true that the elderly group needs to be considered and supported by the community and other decision - making levels. However, it is a group that is not prioritized starting from the family level through community, village and up to the council level. At the community and family level, elderly have been perceived as witches, which may affect the prioritization process…. during the reallocation of funds, it is rare to consider elderly matters, as there are other groups also in need of attention for their health…”- (KI#7).*

### Community awareness of the elderly

The results of this study show that community members are not well aware about the government’s responsibility for elderly healthcare services. This brings some confusion on the responsibilities of the family and community members for the elderly in accessing free healthcare services. This low awareness of the community members about elderly health services makes it difficult for them to understand when it comes to the elderly missing some of the services at the health facilities. For example, when the elderly miss access to medicines while the elderly with NHIF access. At the same time, the central government insists the LGAs supervise the free health services to the elderly without specific funds for the elderly. The community and some of the family members think that the government is responsible for taking care of all the elderly.

*“…sometimes it becomes difficult for the community to contribute to the elderly healthcare services because they know that healthcare services for the elderly are free and the government is responsible for taking care of the elderly...” (KI#16).*

### Less involvement of the CHWs in elderly healthcare services in the community

The participants also reported that the CHWs were supposed to play a great role in the community to help with outreach programs which may include helping the elderly with health problems who need immediate help. The challenge with the CHWs are temporarily engaged by partners on a contract basis in the specific programs in which they are not dealing with elderly healthcare services. This led to less involvement of the CHWs in the elderly matters.

*“...during outreach programs and other community programs implementation, most of the CHWs focus on programs related to pregnant women, HIV, vaccinations, mothers and children. CHWs are less involved in elderly matters due to budget constraints from the government. Another challenge is the scarcity of CHWs in the community, coupled with numerous activities”- (KI#3).*

## Discussion

This study explored the role of CHS on priority setting process to improve health services among the elderly. It highlighted the main findings including community participation on elderly matters, community participation on priority setting of the elderly health services, community perception of the elderly agenda, community awareness about elderly matters and the role of the CHWs on the elderly matters in the community. This study indicate that community members are involved in helping the elderly matters including helping the elderly reach the health facility where necessary, providing information about the elderly who are in need and covering some basic expenses for their health. These results are supported by other studies which indicated that the poor elderly can be helped by the community members where necessary to improve their health [17,19,37–39].

Another finding of this study indicates the importance of CHS during the priority setting process, which plays a significant role in identifying the elderly in need and determining the most necessary health services for them in their communities. This is due to the fact that most of the elderly live in communities where can be identified easily with their health problems and their ability to pay for healthcare services. The Community play a role in identifying these elderly and suggesting the best solutions according to their localities. The findings are consistent with previous studies [13,17,19,40] which indicates the importance of the community in the priority setting process which suggests reasonable measures for the community and simplifies the monitoring and evaluation process according to the community needs especially for the vulnerable people in the community like the elderly.

Furthermore, the study also indicated the community’s perception of the elderly agenda as a challenge. Although there are some community members who perceive that the elderly are important in the community of their wisdom, however, there are some community members who perceive that elderly matters/agenda are not very important to the community since there are so many other health needs to be improved like children’s health programs who are considered to be the next generation and pregnant women. These findings corroborate other studies [41] that indicates the same perceptions from the community that the elderly are not productive in the community.

Another finding of this study revealed that there was community awareness about elderly matters. Most of the community and family members are not well aware of their responsibilities and the government’s responsibilities to the elderly. Most of the community members think that there is only one organ which is responsible for the elderly matters. These findings are supported by other studies [39,42–44] which indicated the same results that family members and community members think that they are not responsible with the elderly healthcare services.

Lastly, the study found that CHWs are less involved. The main challenge with CHWs is that most CHWs are responsible for other health services than elderly matters. This is due to the fact that CHWs are temporarily engaged in specific programs on a contract basis. If they were to be utilized efficiently, they could be very important people in the community who can help with elderly matters and improve the provision of elderly healthcare services. The results corroborate with other studies which indicate the importance of CHWs in a community and how they could be beneficial to the elderly health services [42,45–47]. Most of the studies indicate that CHWs are not well involved in elderly matters due to financial problems at the lower levels with no specific budget to cover their daily expenses since they are not permanently employed [48–51].

### Policy implications

In this study, we acknowledge the fact that some efforts have been made by the government to engage the community in improving the health services for the elderly, including the involvement of CHWs at local levels. Despite all these efforts by the government, there still exist challenges facing the CHS during priority setting process for the improvement of health services for the elderly. One of the challenges is that although some community members perceive that the elderly are important in the community for their wisdom, however, some community members perceive that elderly matters/agenda are not important to the community due to competing with other health needs like children’s health programs. Another challenge was community awareness about elderly matters whereby most of the community and family members are not well aware of their responsibilities and the government’s responsibilities to the elderly. In addition, there is less involvement of the CHWs who are responsible for other temporary health services than elderly matters. The government should improve community engagement, especially CHWs during the priority setting process who are close and aware of the health needs in the community. This can be improved by employing a few CHWs in each village with permanent employment to improve CHS at lower levels. This will help to improve CHS with early identification of the health problems of the vulnerable groups including the elderly for further access to health services. Also, these CHWs will help to provide awareness among community platforms.

## Strengths and limitations of the study

One of the strengths of this study is that it has provided valuable information that will contribute toward improved community participation during the priority setting process to have improved health services for the elderly in rural areas. The study also revealed several challenges facing the CHS including negative perceptions among community members on the elderly agenda, low community awareness of elderly matters in terms of their responsibilities on improving health services of the elderly and low involvement of the CHWs on improving health services of the elderly. The respondents of this study were among people who are responsible for policymaking at the district level which may act as a limitation of the study since such respondents are likely to be defending the ways they run things. They may describe the role of the CHS during priority setting in improving health services for the elderly. However, the findings of the study provided an understanding of the role of CHS during priority setting to have improved healthcare services for the elderly. To address the limitations the study included many participants including members of the different committees, whereby most of them are part of the community.

## Conclusion and recommendations

This study highlighted the role of CHS during the priority setting process to improve health services among the elderly including community participation on elderly matters, community participation on priority setting of the elderly health services, community perception of the elderly agenda, community awareness about elderly matters and the role of the CHWs on the elderly matters in the community. This indicates that the CHS plays a great role during the priority setting process on elderly matters to have improved health services for the elderly including suggesting the best solutions according to their localities. Therefore, the government should involve fully the CHWs to strengthen the CHS which may help the elderly healthcare matters in rural areas to improve elderly healthcare services. In addition, the community have to be educated in order tocreate awareness and positive perception about the elderly agenda and matters through different platforms such as during world elderly day, village meetings, posters, brochures and at the health facility.

## Supporting information

S1 Text Interview guide used in 25th June 2020 08012025. (DOCX)

S2 Text Supporting information. (DOCX)
