# Supplementary material for: Community health systems and priority setting for elderly healthcare services in rural Tanzania: Experience from Nzega and Igunga districts
Source: PLoS One. 2025 Apr 15;20(4):e0321482. doi: 10.1371/journal.pone.0321482 (PMC11999134; doi:10.1371/journal.pone.0321482)
Supplement: S1 Text — (DOCX) [file pone.0321482.s001.docx]

**Community health systems and priority setting for elderly healthcare services in rural Tanzania: Experience from Nzega and Igunga districts.**

**Objective of the study:** This study explored the role of the CHS during priority setting process in improving health services for the elderly in rural Tanzania.

**Appendix I: Interview Guide with the elderly who are committee members at the health facility governing committee/council health service boards on their experience on setting priorities**

**Preliminary things**

- Appointment set
- Venue located

**Before interview**

- Introduction made
- Consent sought

**During interview**

- Attention to details
- Probing at the end of the interview

**At the end of the interview**

- Thank the participant
- Potential of following up questions

**Guiding questions**

1. Thank you very much for giving us your valuable time. Let us begin with your general experience on involvement in ensuring or promoting access of health care services to the elderly population in your facility. **Probes:**
2. *What are your main roles of the community health systems (Involvement in identifying the problem, setting objectives, planning, allocation of resources and overall priority setting, prioritizing elderly issues)?*
3. *How were you engaged for the first time as a community member (orientation to roles, by who and how)?*
4. Thank you very much, I would now like to get your experience on access of health care services by elderly population in your facility. **Probe;**
5. *How the community members help the elderly to reach the health facilities?*
6. *On the number or magnitude of elderly coming to facility for seeking health care services*
7. *What are their main health maters/problems which are solved by the community members?*
8. Thank you, with the health matters facing elderly, I would like to get your experience on how the process of priority setting is carried out. **Probes**
9. *Identification of the problems (who are involved, what happens etc)*
10. *Prioritizing the health needs/problems (How is it done, who does what and how, are there standards for doing this procedure,)*
11. *What challenges are there in identifying and ranking the health problems*
12. *How are the resources allocated to the identified health problems?*
13. *What are the challenges or limitations in allocating the resources?*
14. *What are the facilitators for identifying health problems and allocating resources?*
15. Based on your LGAs knowledge/experience, how are they helpful in setting priorities for the elderly health care services in rural areas? (**Probe**: *How do they operate, why are they helpful or not helpful, what are the steps in setting priorities at the district level?)*
16. How have you been involved in setting priorities for elderly health care services? (**Probe:** *in the community planning process, facility governing committees)*
17. What are the major gaps/weaknesses in setting priorities within your community (**probe**: *at district health service level, health facility, facility governance committees)?*
18. How should priorities including elderly interest be executed in the community **(Probe:** *at district health service level, health facility, facility governance committees*)
19. What general opinion do you have with regards to the efficiency of LGAs for setting priorities in the allocation of resources for the elderly at the district level? **Probe:** *general suggestions and any other comments to have improved health services for the elderly*

***Thank you for your time and co-operation***

**Appendix II: Interview Guide with Decision makers (DPLO, DED, CHMT members) at District Level on setting priorities**

**Preliminary things**

- Appointment set
- Venue located

**Before interview**

- Introduction made
- Consent sought

**During interview**

- Attention to details
- Probing at the end of the interview

**At the end of the interview**

- Thank the participant
- Potential of following up questions

**Guiding questions**

1. Thank you very much for giving us your valuable time. Let us begin with your general experience on involvement in ensuring or promoting access of health care services to the elderly population in your facility. **Probes:**
2. *What are your main roles (Involvement in identifying the problem, setting objectives, planning, allocation of resources and overall priority setting, prioritizing elderly issues)?*
3. *How were you engaged for the first time (orientation to roles, by who and how)?*
4. Now, please tell us your participation in health service governance committee in the health facility and within the community.

**Probe:**

- - - - 1. *His/her involvement in identifying the problem, setting objectives, planning, allocation of resources and overall priority setting, prioritizing elderly issues.*
        2. *Involvement of other people/organs: who are they and what are their roles in identifying the problem, setting objectives, planning, allocation of resources and overall priority setting, prioritizing elderly issues*

1. Asante sana, with the health problems facing elderly, I would like to get your experience on how the process of priority setting is carried out. **Probes**
2. *Identification of the problems (who are involved, what happens etc)*
3. *Prioritizing the health problems (How is it done, who does what and how, are there standards for doing this procedure,)*
4. *What challenges are there in identifying and ranking the health problems*
5. *How are the resources allocated to the identified health problems?*
6. *What are the challenges or limitations in allocating the resources?*
7. *What are the facilitators for identifying health problems and allocating resources?*
8. Based on your knowledge/experience how are Local Government Authorities helpful in setting priorities for the elderly health care services in rural areas? **Probe:**
   - - - 1. *why are they helpful or not helpful?*
         2. *What are the steps on setting priorities at the district level?*
9. In your own words, please tell us what should be done to have a successful decentralized health system for good health care service for the elderly in rural areas. **Probe:**
   - - - 1. *Current health care services for the elderly,*
         2. *Available resources to have improved health care services for the elderly.*
10. How should priorities including elderly interests be executed in the community? **Probe:**
    - - - 1. *at district health service level,*
          2. *At the health facility level (head of the health facility)*
11. What general opinion do you have with regards to the efficiency of LGAs for setting priorities in the allocation of resources for the elderly at the district level? **Probe:** *general suggestions and any other comments to improve health services for the elderly*

***Thank you for your time and co-operation***

**Appendix III: Characteristics of the participants**

| **Basic Participant Information** | |
| --- | --- |
| Region: | Age: |
| District: | Sex: |
| Job Title (optional): | Highest level of Education: |
| Research assistant name: | Participant Code: |
| Work experience: | Experience on working with elderly: |
| Time of the beginning of interview: | Time of finishing the interview: |
| Name of interviewer: | Date of interview: |

**Muongozo wa maswali**

1. Asante kwa kutupatia muda wako kwaajili ya mahojiano nasi. Naomba tuanze mahojiano yetu kwa kupatia uzoefu wako kwa kuhusika kwako katika upatikanaji wa huduma ya afya kwa wazee katika kituo chako cha afya. **Probe:**
2. *Majukumu yako hasa ni nini (katika kuhusika kwako kwenye kutambua matatizo, kupangilia malengo, mipango, ugawanyaji wa rasilimali na kuweka vipaumbele kwa maswala ya kiafya ya wazee)?*
3. *Ulihusishwaje kwa mara ya kwanza (hasa kwenye maelekezo ya majukumu, na nani na kwa namna gani)?*
4. Asante sana, sasa naomba kupata uzoefu wako kwenye upatikanaji wa huduma ya afya kwaajili ya wazee kwenye kituo chako cha kutolea huduma ya afya. **Probe:**
5. *Kwenye idadi ya wazee wanaokuja kwenye kituo cha afya kwauhitaji wa huduma ya afya.*
6. *Matatizo yapi hasa yanayowakumba wazee?*
7. *Kutokana na matatizo hayo yote yanayowapata wazee kunahuduma yoyote maalumu kwaajili ya matatizo hayo?*
8. *Wanapata huduma za matatizo yao (mfano: mashauriano, uchunguzi wa maabara, dawa n.k.)*
9. Asante sana, na shida za kiafya zinazowakabili wazee, napenda kupata uzoefu wako jinsi mchakato wa mpangilio wa kipaumbele unafanywa. **Probe:**
10. *Utambulisho wa matatizo ya wazee (ni akinanani wanaohusika, nini hufanyika nk)*
11. *Kuweka kipaumbele matatizo ya kiafya (inafanywaje, nani hufanya nini na vipi, kuna viwango vya kufanya utaratibu huu,)*
12. *Je, kuna changamoto zipi katika kubaini na kuweka viwango vya matatizo ya kiafya?*
13. *Je, rasilimali zinatengwaje kwa matatizo ya kiafya ya wazee yanayotambuliwa?*
14. *Je, ni changamoto au mapungufu gani katika kugawa rasilimali?*
15. *Je, ni akina nani wanaowezesha utambuzi wa matatizo ya kiafya ya wazee na kugawa rasilimali?*
16. Kulinga na ufahamu/uzoefu wako wa LGAs, ni vipi zinasaidia katika kuweka vipaumbele vya huduma ya afya ya wazee katika maeneo ya vijijini? (**Probe:** *Je, zinafanyaje kazi, kwanini zinasaidia au hazina msaada, ni hatua gani za kuweka vipaumbele katika ngazi ya wilaya?)*
17. Je, umeshiriki vipi katika kuweka vipaumbele vya huduma za afya za wazee? (**Probe:** *katika mchakato wa upangaji wa jamii, kamati za usimamizi za kituo)*
18. Je, ni mapungufu / udhaifu gani mkubwa katika kuweka vipaumbele ndani ya jamii yako (**probe**: *kwa huduma za afya katika ngazi ya wilaya, kituo cha afya, kamati za usimamizi wa kituo)?*
19. Vipi vipaumbele pamoja na mambo ya wazee vinapaswa kutekelezwa katika jamii (**Probe:** *kwa huduma za afya katika ngazi ya wilaya, kituo cha afya, kamati za usimamizi wa kituo*)
20. Je, kwa ujumla una maoni gani kuhusu ufanisi wa mamlaka ya serikali za mitaa (halmashauri) kwa kuweka vipaumbele katika mgawanyo wa rasilimali kwa wazee katika ngazi ya wilaya? **Probe:** *maoni yako kwa ujumla katika kuboresha huduma za afya kwa wazee.*

***Asante kwa muda na ushirikiano wako***

**Muongozo wa maswali**

1. Asante sana kwa kutupatia furusa hii ya kuongea nasi. Tuanze na uzoefu wako kwa ujumla juu ya kuhusika katika kuhakikisha au kukuza upatikanaji wa huduma za afya kwa wazee katika kituo chako cha afya. Probe:
2. *Je, majukumu yako makuu ni nini (Kushiriki katika kutambua matatizo, kuweka malengo, kupanga, ugawaji wa rasilimali na mpangilio mzima wa vipaumbele, kuweka kipaumbele katika maswala ya wazee)?*
3. *Je, ulihusika vipi kwa mara ya kwanza (kuelekezwa katika majukumu, na nani na namna gani ulielekezwa)?*
4. Sasa, tafadhali tuambie ushiriki wako katika kamati ya usimamizi wa huduma za afya katika kituo cha afya na ndani ya jamii. Probe:
5. *Kuhusika kwake katika kubaini matatizo/shida, kuweka malengo, kupanga, ugawaji wa rasilimali na mpangilio wa vipaumbele kwa jumla, kuweka kipaumbele maswala ya afya ya wazee.*
6. *Kuhusika kwa watu / vyombo vingine: ni akina nani na ni nini majukumu yao katika kutambua shida/matatizo, kuweka malengo, kupanga, ugawaji wa rasilimali na mpangilio wa kipaumbele kwa jumla, na kuweka mbele maswala ya wazee*
7. Asante sana, kutokana na matatizo/shida za kiafya zinazowakabili wazee, napenda kupata uzoefu wako jinsi mchakato wa mpangilio wa vipaumbele unavyofanywa. Probe:
8. *Utambulisho wa shida za kiafya za wazee (akina nani wanahusika, nini hufanyika nk)*
9. *Kuweka kipaumbele matatizo ya kiafya ya wazee (inafanywaje, nani hufanya nini na vipi, kuna viwango vya kufuata katika kufanya utaratibu huu,)*
10. *Je, kuna changamoto gani katika kubaini na kuweka viwango vya matatizo ya kiafya za wazee?*
11. *Je, rasilimali zinatengwaje kwa matatizo ya kiafya za wazee yanayotambuliwa?*
12. *Je, ni changamoto au mapungufu gani katika kugawa rasilimali?*
13. *Je, ni wawezeshaji gani wa kutambua shida za afya za wazee na kugawa rasilimali?*
14. Kwa uzoefu wako jinsi gani Mamlaka ya Serikali za Mitaa zinasaidia kuweka vipaumbele vya huduma ya afya ya wazee katika maeneo ya vijijini? Probe:
15. *kwanini zinasaidia au sio msaada?*
16. *Je, ni hatua gani za kuweka vipaumbele katika ngazi ya wilaya?*
17. Kwa maneno yako mwenyewe, tafadhali tuambie nini kifanyike kuwa na mfumo mzuri wa kiafya wa huduma bora kwa huduma ya afya kwa wazee katika maeneo ya vijijini. Probe:
18. *Huduma za afya za wazee kwa sasa,*
19. *Kwa kutumia rasilimali zinazopatikana katika kuboresha huduma za afya kwa wazee.*
20. Jinsi gani vipaumbele vyenye masilahi ya wazee vinapaswa kutekelezwa katika jamii? Probe:
21. *katika ngazi ya huduma ya afya ya wilaya,*
22. *Katika kiwango cha kituo cha afya (mkuu wa kituo cha afya)*
23. Je, una maoni gani kwa ujumla kuhusu ufanisi wa mamlaka ya serikali za mitaa (Halmashauri) kwa kuweka vipaumbele katika mganyo wa rasilimali katika huduma za afya kwa wazee katika ngazi ya wilaya? **Probe:** *maoni katika kuboresha huduma za afya kwa wazee*

*Asante kwa wakati wako na ushirikiano*
